# Chronic Pesticide Exposure in Farm Workers Is Associated with the Epigenetic Modulation of hsa-miR-199a-5p

**DOI:** 10.3390/ijerph19127018

**Published:** 2022-06-08

**Authors:** Giuseppe Gattuso, Luca Falzone, Chiara Costa, Federica Giambò, Michele Teodoro, Silvia Vivarelli, Massimo Libra, Concettina Fenga

**Affiliations:** 1 Department of Biomedical and Biotechnological Sciences, University of Catania, 95123 Catania, Italy; peppeg9305@gmail.com (G.G.); m.libra@unict.it (M.L.); 2 Epidemiology and Biostatistics Unit, Istituto Nazionale Tumori IRCCS Fondazione G. Pascale, 80131 Naples, Italy; 3 Clinical and Experimental Medicine Department, University of Messina, 98125 Messina, Italy; ccosta@unime.it; 4 CEMAD Digestive Disease Center, Fondazione Policlinico Universitario “A. Gemelli” IRCCS, Università Cattolica del Sacro Cuore, 00168 Roma, Italy; federica.giambo@guest.policlinicogemelli.it; 5 Department of Biomedical and Dental Sciences and Morphofunctional Imaging, Occupational Medicine Section, University of Messina, 98125 Messina, Italy; michele.teodoro@unime.it (M.T.); silvia.vivarelli@unime.it (S.V.); concettina.fenga@unime.it (C.F.); 6 Research Centre for Prevention, Diagnosis, and Treatment of Cancer, University of Catania, 95123 Catania, Italy

**Keywords:** pesticide exposure, microRNAs, liquid biopsy, ddPCR, hsa-miR-199a-5p, epigenetics

## Abstract

The increasing use of pesticides in intensive agriculture has had a negative impact on human health. It was widely demonstrated how pesticides can induce different genetic and epigenetic alterations associated with the development of different diseases, including tumors and neurological disorders. Therefore, the identification of effective indicators for the prediction of harmful pesticide exposure is mandatory. In this context, the aim of the study was to evaluate the modification of hsa-miR-199a-5p expression levels in liquid biopsy samples obtained from healthy donors and farm workers with chronic exposure to pesticides. For this purpose, the high-sensitive droplet digital PCR assay (ddPCR) was used to detect variation in the expression levels of the selected microRNA (miRNA). The ddPCR analyses revealed a significant down-regulation of hsa-miR-199a-5p observed in individuals exposed to pesticides compared to control samples highlighting the good predictive value of this miRNA as demonstrated by statistical analyses. Overall, the obtained results encourage the analysis of miRNAs as predictive biomarkers of chronic pesticide exposure thus improving the current strategies for the monitoring of harmful pesticide exposure.

## 1. Introduction

Pesticides are defined as a substance or mixture of substances used to prevent, repel, and destroy different pests including insects, weeds, microorganisms, and disease vectors to protect plants and humans [1,2]. According to the data published by the Food and Agriculture Organization of the United Nations (FAO) in 2020, the use of pesticides increased from 1.54 kg to 2.63 kg per hectare from 1990 to 2017, with a total annual pesticide use increasing from 2.29 million tons in 1990 to 4.11 million tons in 2017 [3]. Moreover, pesticides are widely used in agriculture to protect different types of fruits and vegetables, some of which are studied for their nutraceutical effects in different pathologies including tumors [4,5,6,7,8]. However, increasing use of pesticides has been observed in the last years also in other environments. Indeed, in addition to their common use, pesticides are also used in parks, forestry, athletic fields, industrial sites, educational lawn care facilities, industrial vegetation control, and as public health measures thus increasing the environmental exposure to these substances [9].

Interestingly, in the United States, herbicides account for 50% of the top ten active chemicals used by non-agricultural professional pesticide users (insecticides for 40% and fungicides for 10%). Regarding the use of pesticides in agriculture, herbicides account for 48%, followed by fungicides (16%), insecticides (8%), and plant growth regulators (8%) [10]. In Europe, the primary pesticide categories with the biggest sales volumes in 2011 and 2019 were fungicides and herbicides, and moss killers. In particular, four European countries, specifically Germany, Spain, France, and Italy, recorded the largest volumes of pesticide sales [11]. Currently, several studies on the effects of pesticides have been performed among pesticide users, highlighting a strong association between pesticide exposure and increased risk of different types of tumors such as skin, lung, breast, ovarian, testicular, and prostatic cancer, as well as Non-Hodgkin Lymphoma (NHL), multiple myelomas, and leukemia [12,13,14,15]. Moreover, reproductive disorders or neurological diseases such as Parkinson’s disease (PD), Alzheimer’s disease (AD), and amyotrophic lateral sclerosis, have also been associated with pesticide exposure [16,17].

These data indicate that pesticides can cause detrimental effects on human health through epigenetic modifications [18,19]. Specifically, recent evidence suggests that exposure to toxic compounds influences the expression levels of microRNAs (miRNAs), known to be involved in the development of various diseases [20]. Notably, miRNAs are a class of short non-coding RNAs with a length of 18–25 nucleotides that play an active role in epigenetic regulation of gene expression and are also involved in post-transcriptional gene silencing [21]. miRNAs can be easily detected both intracellularly and extracellularly with the possibility to detect these molecules also in different biological fluids including serum, plasma, saliva, urine, and others [22,23,24]. Hence, miRNAs could be used as indicators for specific chemicals and their alterations could be predictive of the development of human diseases [25,26,27].

Our research group has recently identified genetic and epigenetic alterations induced by pesticide exposure by analyzing publicly available datasets on pesticide exposure. Through these analyses, a panel of 20 miRNAs significantly dysregulated as a consequence of pesticide exposure were identified. Further computational approaches demonstrated that the identified miRNAs are involved in the alteration of key cellular and molecular pathways associated with different human diseases [28].

Other studies have identified several miRNAs whose expression levels were significantly dysregulated as a consequence of pesticide exposure. Li and colleagues identified 30 miRNAs significantly dysregulated in porcine kidney epithelial PK15 cells after the exposure to the insecticide dichlorvos; among these, 16 were significantly up-regulated and 14 were significantly down-regulated [29]. An in vivo study has been conducted by Wang and colleagues to evaluate the effect of two insecticides (fipronil and triazophos) on miRNAs expression in zebrafish. The authors demonstrated that miR-21, miR-31, miR-203b, and miR-455 levels were increased in exposed animals, while miR-135c, miR-30b, and miR-365 levels decreased after treatment with triazophos [30].

All these studies suggest that the analysis of miRNAs can be useful to predict the risk of pesticide exposure representing novel non-invasive biomarkers for the early diagnosis of pesticide-related diseases. On these bases, the expression levels of hsa-miR-199a-5p were evaluated in liquid biopsy samples obtained from farm workers chronically exposed to pesticides and healthy donors with indoor work activities without occupational or environmental exposure to chemicals. Of note, hsa-miR-199a-5p has been associated with different neoplastic and neurodegenerative diseases [31,32,33,34]. In addition, two recent studies described the dysregulation of hsa-miR-199a-5p due to the exposure to toxic compounds derived from pesticides including 1-Methyl-4-Phenylpyridinium (MPP+) [34,35]. Despite these few findings on the dysregulation of hsa-miR-199a-5p, no conclusive studies on the alteration of the circulating levels of this miRNA in individuals exposed to pesticides have been performed. Therefore, in the present study, the expression levels of hsa-miR-199a-5p were investigated in a pilot cohort of 28 farm workers chronically exposed to pesticides and in nine healthy donors by using the high-sensitive droplet digital PCR (ddPCR) to evaluate the modulation of hsa-miR-199a-5p expression.

## 2. Materials and Methods

### 2.1. Sample Collection

In order to evaluate the expression levels of hsa-miR-199a-5p, 37 subjects were recruited for this study. In particular, 28 liquid biopsy samples from farm workers chronically exposed to pesticides and nine samples obtained from healthy donors were analyzed. The nine healthy donors were selected among physicians or indoor workers not occupationally exposed to pesticides. All the samples were collected during routine blood testing or an occupational medicine surveillance program. For each subject, two blood samples were collected in different test tubes to obtain, respectively, serum and plasma samples by centrifugation at 2000× *g* for 10 min at room temperature. Buffy coat and red cells were also collected and stored at −80 °C. All the subjects enrolled in this study met specific exclusion criteria; in particular, individuals with a history of previous/concomitant cancers, chronic inflammatory diseases, or neurodegenerative disorders were excluded. All the relevant features of both farm workers and healthy donors are reported in Table 1.

### 2.2. Extraction of miRNAs and cDNA Retrotranscription

The circulating miRNAs were extracted from the serum samples of cases and controls as already described [36]. Briefly, serum samples were centrifuged at 2000× *g* 10 min at room temperature to remove debris and protein aggregates contained in serum samples. After centrifugation, total RNA and miRNAs were isolated from 200 µL of samples by using the miRNeasy Serum/Plasma Kit (Cat. No. 217184, Qiagen, Hilden, Germany) according to the manufacturer’s instructions. In order to normalize the circulating levels of hsa-miR-199a-5p, the exogenous UniSp4 spike-in control (Cat. No. 339390, Qiagen, Hilden, Germany) was used in each sample during the extraction procedure. Finally, 4 µL of miRNAs were retrotranscribed into cDNAs using the miRCURY LNA RT Kit (Cat. No. 339340, Qiagen, Hilden, Germany).

### 2.3. Analysis of miRNAs Expression Levels through Droplet Digital PCR

The expression levels of hsa-miR-199a-5p were evaluated in liquid biopsy samples by using the ddPCR system. In particular, the ddPCR reaction mix was generated by using 11 µL of 2x QX200™ ddPCR™ EvaGreen Supermix (Cat. No. 1864034, Bio-Rad Laboratories, Inc., Hercules, CA, USA), 1.1 μL of miRNA-specific primer miRCURY LNA miRNA PCR Assay (Cat. No. 339306; UNISP4 Cat. No. YP00203953; hsa-miR-199a-5p Cat. No. YP00204494, Qiagen, Hilden, Germany), 6.9 μL of RNase and DNase free-water, and 3 μL of cDNA for a total 22 µL. Then, 20 µL of reaction was used to generate almost 20,000 droplets by using the X200™ Droplet Generator (Cat. No. 1864002, Bio-Rad Laboratories, Inc., Hercules, CA, USA). Subsequently, the droplets were amplified in a 96-well plate through the C1000 Touch™ Thermal Cycler (Bio-Rad Laboratories, Inc., Hercules, CA, USA). The amplification was performed according to the following thermal conditions: Taq activation at 95 °C for 5 min followed by 40 cycles at 95 °C for 30 sec and 56 °C for 1 min for the denaturation and annealing/extension, respectively, signal stabilization at 4 °C for 5 min and 90 °C for 5 min followed by an infinite hold at 4 °C. Then, the 96-well plate was loaded into the QX200 Droplet Reader (Bio-Rad Laboratories, Inc., Hercules, CA, USA) to read the positive and negative droplets obtained for each sample analyzed. The exogenous spike-in control UniSp4 was used to normalize the copies/μL of hsa-miR-199a-5p to avoid possible bias in miRNA quantification due to errors in the extraction procedure.

### 2.4. Statistical Analyses

The absolute quantification of hsa-miR-199a-5p circulating levels was obtained by using the QuantaSoft software (Bio-Rad Laboratories, Inc., Hercules, CA, USA). The distribution of miRNA expression levels was evaluated by using the Kolmogorov–Smirnov normality test. The Mann–Whitney and Kruskal–Wallis tests were performed to evaluate the statistical difference of hsa-miR-199a-5p between farm workers and healthy donors or among different stratification groups of workers. In addition, a contingency table was obtained by analyzing exposed and non-exposed individuals according to their expression levels of hsa-miR-199a-5p; the statistical significance was calculated through the Fisher’s exact test. Despite the limited number of individuals recruited, the preliminary specificity and sensitivity rates of hsa-miR-199a-5p were also evaluated through Receiver Operating Characteristic (ROC) curves in order to establish the diagnostic potential of the selected miRNA as already described [36,37]. All statistical analyses were performed by using GraphPad Prism v. 8.

## 3. Results

### 3.1. Clinical Features and Time of Exposure of Farm Workers

During the collection of biological samples, various socio-demographic, anamnestic, and clinical data were collected for each individual enrolled in the study. These data are schematically reported in Table 1. All the farm workers enrolled in this study were chronically exposed to different pesticides. In particular, 17 women and 11 men were enrolled and were equally distributed according to their age; age ≤ 45 years old and subjects with age > 45 years old. All the farmers included in the study had a working history of at least three years with an average annual working activity with pesticides of about 101 days. During their professional activity, all workers constantly used personal protective equipment (PPE); in particular, 10 individuals used washable PPE and 17 disposable PPE. Finally, data related to cigarette smoking were collected, showing that 12 of the 28 farm workers were constantly using tobacco with an average of about 13 cigarettes per day. Tobacco smoking does not represent a confounding factor as demonstrated by analyzing the expression levels of hsa-miR-199a-5p in smoker and non-smoker workers. In addition, a similar percentage of smokers was also included in the control group. Therefore, no statistical differences in the composition of cases and control groups exist regarding smoking habits.

### 3.2. Analysis of miRNA Expression Levels in Liquid Biopsy Samples of Farm Workers Chronically Exposed to Pesticides and Healthy Controls

**Figure 1:**
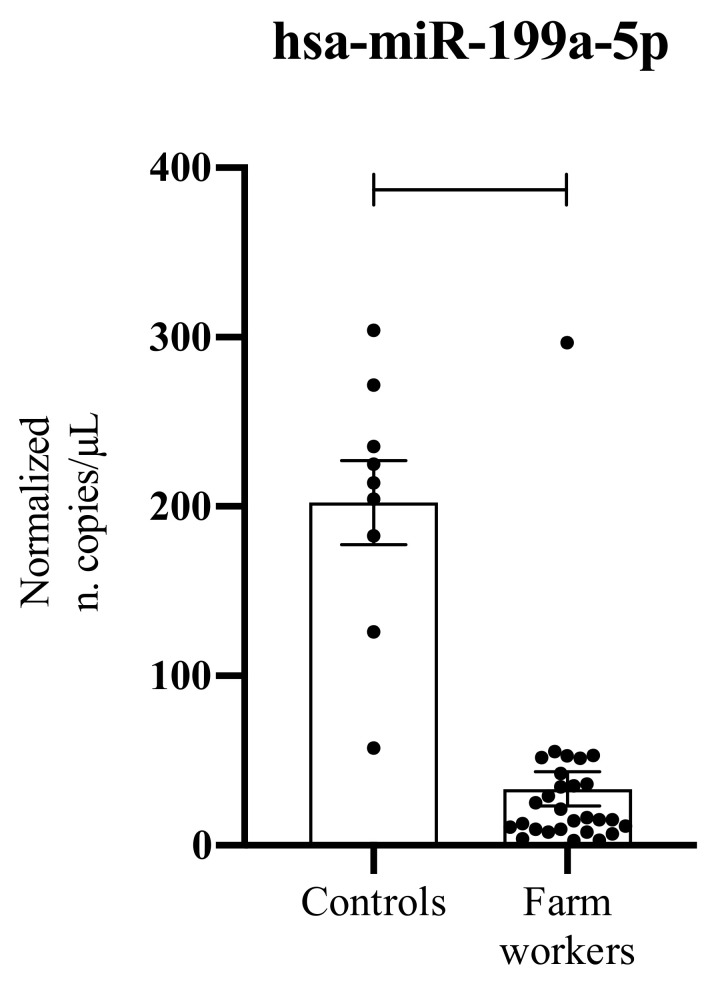
Serum levels of hsa-miR-199a-5p in normal controls and farm workers chronically exposed to pesticides. ****: *p* < 0.0001.

The ddPCR analysis conducted on 37 serum samples demonstrated a strong down-regulation of hsa-miR-199a-5p in liquid biopsy samples obtained from farm workers compared to those obtained in healthy subjects (Figure 1). Although performed in a low number of samples, statistically significant results were obtained (*p* < 0.0001) (Figure 1).

With regards to farm workers, additional stratification analyses were performed considering some workers’ features such as gender, age, cigarette smoking, employee seniority, annual pesticide exposure, and the use of PPE. The results revealed that hsa-miR-199a-5p expression levels do not vary significantly stratifying the workers according to these categories. These data suggest that hsa-miR-199a-5p modulation is independent of cigarette smoking or other parameters (Supplementary Figure S1).

### 3.3. Diagnostic Value of hsa-miR-199a-5p

**Figure 2:**
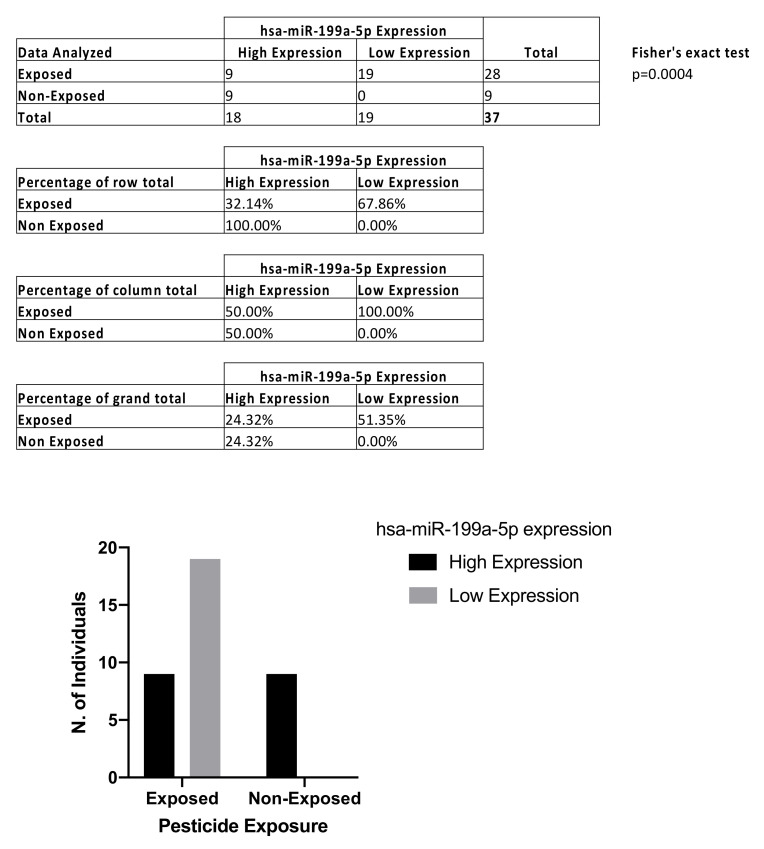
Contingency table and Fisher’s exact test to establish the association between hsa-miR-199a-5p and pesticide exposure.

The ddPCR analysis performed on liquid biopsy samples showed significant downregulation of circulating levels of hsa-miR-199a-5p in farm workers chronically exposed to pesticides compared to healthy controls. Analyzing farm workers and healthy donors as a single group, the median value of hsa-miR-199a-5p expression was calculated and used as the cut-off value to divide the entire cohort into hsa-miR-199a-5p high-expression and low-expression groups. The median normalized value of hsa-miR-199a-5p expression was 34.52 copies/μL and this cut-off was used to obtain the contingency table. More in detail, contingency analysis through Fisher’s exact test and ROC analysis were performed to evaluate the diagnostic value of the investigated miRNA and its association with pesticide exposure. Fisher’s exact test showed that there is a statistically significant association between high hsa-miR-199a-5p expression levels and pesticide exposure (*p* < 0.004; Figure 2).

Despite the limited number of samples analyzed, ROC analysis confirmed these data showing high sensitivity and specificity rates of hsa-miR-199a-5p (96.43% and 100% respectively) with an AUC = 0.9683; 95% CI 0.9063 to 1.000 (*p* < 0.0001) (Supplementary Figure S2).

## 4. Discussion

A steady increase in the use of pesticides has been observed over the past few decades and global pesticide production is expected to increase in the near future. Unfortunately, it is also known that their chemical residues can have a negative impact on human health [2].

At present, many studies have associated occupational pesticide exposure with an increased risk of developing cancers and neurological disorders [16,17,38,39,40]. Epigenetic mechanisms, including DNA methylation, gene dysregulations, and the alteration of miRNA expression levels could be the underlying mechanisms through which pesticides cause adverse effects in humans [28]. However, a clear association between pesticide exposure and epigenetic alterations, especially miRNA dysregulation, has not been identified yet nor reliable biomarkers associated with pesticide exposure are currently available. Therefore, there is a consistent need to identify potential biomarkers predictive of chronic exposure to pesticides.

For this purpose, the circulating expression levels of hsa-miR-199a-5p were investigated in a pilot cohort of individuals exposed to pesticides and healthy controls using ddPCR.

The miR-199a-5p is a member of the highly conserved miR-199 family, which consists of miR-199a and miR-199b. Both miRNAs are involved in several processes such as cell death and cell survival [41]. miR-199a-3p and miR-199a-5p, originating from chromosome 1 and chromosome 19, respectively, are both mature forms of hsa-miR-199a. Moreover, there are also two mature forms of hsa-miR-199b: miR-199b-5p and miR-199b-3p [42].

In the present study, miR-199a-5p was selected as a putative indicator of chronic pesticide exposure potentially associated with the development of different pathologies known to be associated with exposure to different chemicals, including pesticides [43,44,45,46,47,48].

In order to validate the putative value of hsa-miR-199a-5p in predicting the risk of chronic pesticide exposure, the circulating levels of this miRNA were investigated on 28 serum samples from farm workers chronically exposed to pesticides compared with the expression levels observed in nine healthy donors. Since miRNAs could be expressed in low concentrations in liquid biopsy samples, the high-sensitive ddPCR assay was used.

The ddPCR results revealed that the expression levels of circulating hsa-miR-199a-5p were significantly down-regulated in the group of farm workers chronically exposed to pesticides compared to healthy controls (*p* < 0.0001).

Despite the lower number of samples analyzed, hsa-miR-199a-5p demonstrated great accuracy in correctly identifying subjects exposed to pesticides. Indeed, the ROC curve showed a sensitivity and a specificity rate ranging from 95% to 100%. Thus, the ddPCR evaluation of hsa-miR-199a-5p expression levels in serum samples may be useful to predict the risk of chronic pesticide exposure.

Recently, hsa-miR-199a-5p was found to serve as a tumor suppressor and to be downregulated in several cancers, including breast and colorectal cancer [49,50,51]. Other studies showed altered expression levels of miR-199a-5p in lung and bladder cancers as well [47,52]. Other studies showed a significant decrement of hsa-miR-199a-5p in neurological disorders as the miRNA was found down-regulated in Parkinson’s disease as well as in patients with amyotrophic lateral sclerosis [33,53].

As demonstrated in previous studies, ddPCR represents an extremely high sensitivity technique for the analysis of liquid biopsy samples, which can detect low expressed targets, such as circulating DNA and circulating miRNAs associated with tumors as well as low amounts of viral and bacterial nucleic acids [36,54,55,56].

The results obtained herein, coupled with the literature data reporting a clear association between pesticide exposure and increased risk of developing neoplastic or degenerative diseases, encourage the use of both ddPCR and liquid biopsy for the monitoring of the health status of workers occupationally exposed to pesticides. Therefore, the ddPCR analysis of hsa-miR-199a-5p circulating levels could improve the current surveillance strategies for workers at risk for pesticide exposure by developing novel diagnostic strategies for both the prediction of harmful pesticide exposure and the early diagnosis of pesticide-related diseases.

The present study presents several limitations mainly represented by the low number of samples analyzed and the evaluation of hsa-miR-199a-5p as a single miRNA potentially dysregulated by chronic exposure to pesticides. As a consequence, this pilot study represents only the starting point for a further in-depth investigation of pesticide exposure potentially associated with the development of both tumor and neurodegenerative diseases. Further experiments are needed to validate the preliminary findings here obtained in order to propose hsa-miR-199a-5p as an effective biomarker of pesticide exposure.

## 5. Conclusions

Overall, a significant down-regulation of hsa-miR-199a-5p was observed in farm workers chronically exposed to pesticides. To the best of our knowledge, this is the first study investigating the circulating levels of hsa-miR-199a-5p as a potential biomarker of pesticide exposure. The statistically significant results obtained encourage further investigation on this miRNA that will be performed on farm workers observed during the time. In addition, further investigations on hsa-miR-199a-5p mRNA targets and other miRNAs are needed to identify an altered miRNA signature associated with chronic pesticide exposure and the molecular pathways modulated by these putative miRNAs.

## Figures and Tables

**Table 1:** Clinical features and time exposure of farm workers chronically exposed to pesticides. * Missing data.

Subjects’ Characteristics	Farm Workers (N. 28)		Healthy Donors (N. 9)	
	N.	%	N.	%
**Gender**				
Male	11	39.29	8	88.89
Female	17	60.71	1	11.11
**Age**				
≤45	13	46.43	0	0
45–59	8	28.57	5	55.56
≥0	7	25.00	4	44.44
**Cigarette Smoking**				
Yes	12	42.86	4	44.44
No	16	57.14	5	55.56
Employee Seniority (years) *				
≤5	9	34.62	/	
5–9	10	38.46	/	
≥10	7	26.92	/	
**Annual Pesticide Exposure (days)**				
<50	5	17.86	/	
50–100	6	21.43	/	
>100	17	60.71	/	
**PPE**				
Disposable	17	60.71	/	
Washable	11	39.29	/	

## Data Availability

The data generated in this study were deposited in the Zenodo platform (https://doi.org/10.5281/zenodo.6504557) (created and accessed on 29 April 2022) and are also available from the corresponding author upon reasonable request.

## Supplementary Materials

The following supporting information can be downloaded at: https://www.mdpi.com/article/10.3390/ijerph19127018/s1, Figure S1: Analysis of hsa-miR-199a-5p expression levels in farm workers stratified according to different characteristics. (A) Stratification by gender (Mann–Whitney test); (B) Stratification by age (Mann–Whitney test); (C) Stratification by cigarette smoking (Mann–Whitney test); (D) Stratification by employee seniority (Kruskal–Wallis test); (E) Stratification by annual pesticide exposure (Kruskal–Wallis test); (F) Stratification by use of PPE (Mann–Whitney test). No significant data were obtained; Figure S2: ROC analysis demonstrated the high diagnostic value of hsa-miR-199a-5p (96.43% sensitivity and 100% specificity).
