# Supplementary material for: Chronic Pesticide Exposure in Farm Workers Is Associated with the Epigenetic Modulation of hsa-miR-199a-5p
Source: Int J Environ Res Public Health. 2022 Jun 8;19(12):7018. doi: 10.3390/ijerph19127018 (PMC9222590; doi:10.3390/ijerph19127018)
Supplement: Supplementary file 1 [file ijerph-19-07018-s001.zip › ijerph-1736122-supplementary.pdf]

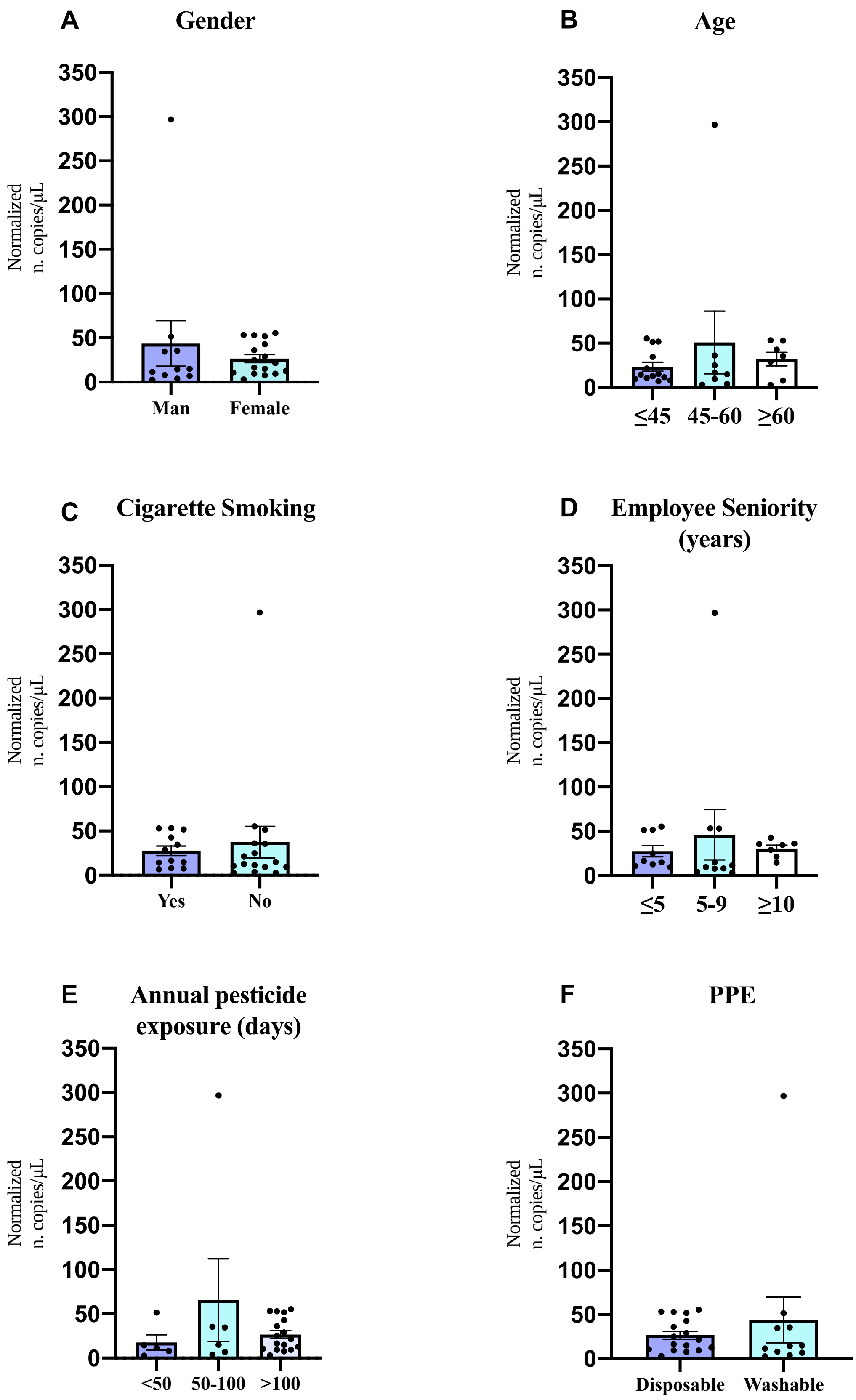

Supplementary Figure S1. Analysis of hsa-miR-199a-5p expression levels in farm workers stratified according different characteristics. (**A**) Stratification by gender (Mann-Whitney test); (**B**) Stratification by age (Mann-Whitney test); (**C**) Stratification by cigarette smoking (Mann-Whitney test); (**D**) Stratification by employee seniority (Kruskal-Wallis test); (**E**) Stratification by annual pesticide exposure (Kruskal-Wallis test); (**F**) Stratification by use of PPE (Mann-Whitney test). No significant data were obtained.

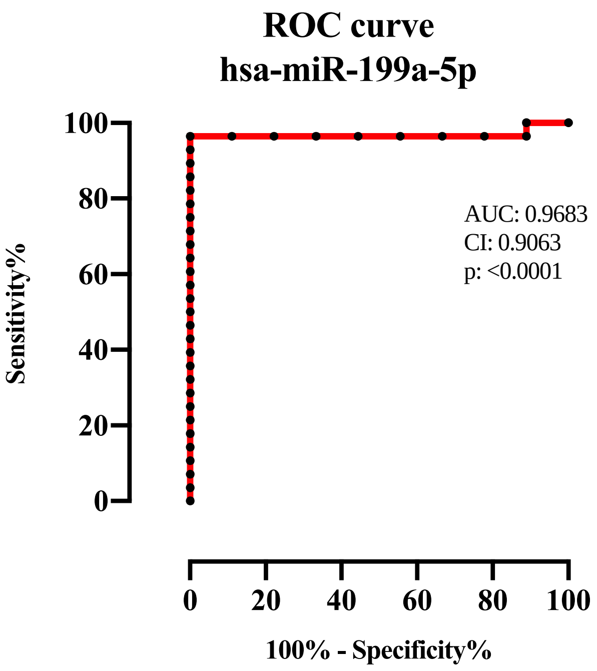

Supplementary Figure S2. ROC analysis demonstrated the high diagnostic value of hsa-miR-199a-5p (96.43% sensitivity and 100% specificity).
